# Chiseling the Mastoid Process

**Published:** 1884-11

**Authors:** 


### Editorial.

---

### Chiseling the Mastoid Process.

Dr. A. Hartman, of Berlin, details (*Arch. of Otol.*) the histories of fourteen cases of abscess of the mastoid process which were treated by him at his polyclinic, and which required opening by operation. Of the fourteen cases, the mastoid disease followed acute suppuration of the middle ear in four, and chronic suppuration of the middle ear or its complications in ten. Of the ten cases which resulted from chronic suppuration, sequestra had formed in four, caries in four, polypi in the middle ear and mastoid in one, and cholesteatoma in an enlarged mastoid antrum in one. Death resulted in two cases, but in one facial paralysis and symptoms of meningitis had developed before the operation, and in the other pyæmia and thrombus were present and the operation was proposed as a last resource.

Concluding a full report of each of the fourteen cases, Dr. Hartman presents a short general review of his practical experience, utilizing his former observations in operating upon the mastoid, which is of great interest. As to the choice of the point of operation, he says: "The incision of the skin and the opening of the bone should be practiced at the line of attachment of the auricle, or, at least, immediately behind it." The mastoid should be opened directly beneath this, when pus may be reached at a trifling depth. In cases where fistulas have formed, it is likewise most judicious to so incise the skin that the bone beneath the line of attachment of the auricle or immediately beneath it may come into the region to be chiseled.

The reader is warned against the danger of wounding the transverse sinus or penetrating the middle fossa of the cranium. According to the results of examinations made by the author upon the cadaver, which are essentially the same as those of Bezold and others, "a sharp forward curve of the transverse sinus toward the posterior wall of the auditory canal is very frequent," the distance between these two points in 100 temporal bones, amounting, in 41 cases, to one centimetre or less; in one case it being only five millimetres; in five cases, six millimetres, and in six cases, seven millimetres. The greatest measurement found was nineteen millimetres, while the average in the whole one hundred was eleven and five-tenths millimetres. The danger of wounding the transverse sinus is best seen on horizontal section of the temporal bone.

In view of the danger of opening the middle cranial fossa, he adheres to the rule previously laid down by him, that the operation-canal should not extend higher than the level of the upper wall of the auditory canal, as his anatomical investigations have shown that the floor of the middle fossa of the cranium is not infrequently separated from the upper wall of the auditory canal by only a thin, long lamella, and lies but a little above it. In his operations upon the cadaver, after the manner of Buck, who sets the drill a little above the line of the external auditory canal and penetrates inward and a little upward and forward, he penetrated the middle cranial fossa with the drill in three cases out of one hundred.

"Those," he says, "who are familiar with the anatomical relations of the parts will, therefore, avoid the use of drills or trephines, such as are employed by many physicians. When, in using such an instrument, the sinus is chanced upon, an injury to it is inevitable; whereas, in the operation with the chisel, as the ground is kept clear for inspection, we can recognize the danger in time and avoid it."

In operating, both the incision through the skin and the bony canal chiseled out, should be made large, so that a free inspection of the wound-cavity may be possible during the after-treatment, so that pieces of bone which may become detached later, may be easily removed, and so that any remaining or luxuriant granulations may be readily destroyed with caustics or removed with a sharp spoon. The operation-canal should be kept open by rubber tubes immediately after the operation, to be replaced later by thick, and later still by thin lead ones, converting the lead tube into a sort of funnel-shape, by rounding one end with a knife and splitting the other end, and bending the two halves apart. Old retained secretions or cholesteatomatous masses in the mastoid should also be completely removed, which is often impossible, except with the author's inflexible tympanum tube.

To prevent inflammatory reaction after the operation, he covers the walls of the cavity and of the canal in the bone with iodoform, powdered. In the cases with previous acute symptoms, there followed an immediate recedence of them and a surprisingly rapid cure. He considers that possibly the favorable effects of the remedy depends upon the circumstance that it forms with the underlying tissues a soft, firmly attached crust. By this crust-formation, on account of the scanty secretions, the first dressing can be left unchanged for two or three days.

Considering, on the one hand, that the artificial opening of the mastoid in acute as well as chronic diseases leads to a prompt and perfect cure, and that the operation must be regarded as entirely free from danger; and, on the other hand, the often very tedious, incomplete recoveries, accompanied, perhaps, with severe functional disturbances and the danger also of extension of the disease to contiguous and more vital structures by the conservative methods of treatment, a decision must be given in favor of the former operative plan. Again, sequestra may exist, which cannot be determined, but which can only be removed by an operative procedure.

